# Efficacy and patient selection of consolidative thoracic radiotherapy following chemoimmunotherapy in ES-SCLC

**DOI:** 10.3389/fcell.2026.1850317

**Published:** 2026-06-11

**Authors:** Donghong Yang, Ruqi Chen, Tongtong Xia, Guohu Zhang, Junhong Jiang, Guanbin Huang, Yiping Luo, Muwen Lin, Xiaolin Li, Dongxiao Yang, Yuying Chen, Hualin Chen

**Affiliations:** 1 Department of Oncology, Affiliated Hospital of Guangdong Medical University, Zhanjiang, Guangdong, China; 2 Department of Pulmonary Oncology, Affiliated Hospital of Guangdong Medical University, Zhanjiang, Guangdong, China; 3 The First Clinical Medical School, Guangdong Medical University, Zhanjiang, Guangdong, China; 4 Department of Oncology, Southern Theater Command Navy First Hospital, Zhanjiang, Guangdong, China

**Keywords:** chemoimmunotherapy, consolidative thoracic radiotherapy, ES-SCLC, patient selection, survival

## Abstract

**Background:**

Despite significant progress with immunotherapy in extensive-stage small-cell lung cancer (ES-SCLC), progression-free survival (PFS) remains limited, and relapse is common. Whether consolidative thoracic radiotherapy (cTRT) administered after first-line chemoimmunotherapy offers additional clinical benefit remains uncertain, and the lack of clear patient selection criteria further restricts its application in real-world.

**Methods:**

ES-SCLC patients who received first-line PD-1/PD-L1 inhibitor plus platinum-etoposide and achieved disease control were included. All patients were divided into two groups based on whether they received cTRT. The primary endpoint was PFS. Secondary endpoints included overall survival (OS), depth of response (DpR), and safety.

**Results:**

A total of 91 patients were included. DpR analysis showed a higher incidence of additional tumor shrinkage in the cTRT group (*P* = 0.007). Patients who received cTRT had significantly longer unadjusted median PFS and median OS (log-rank test *P* < 0.001). After weighting, survival differences were attenuated but remained numerically in favor of the cTRT group. Cox analysis showed receipt of cTRT was independently associated with prolonged PFS (*P* = 0.036) and OS (*P* = 0.033). Logistic regression revealed that poorer ECOG PS (*P* = 0.047) and higher clinical stage (*P* = 0.046) were independently associated with a lower likelihood of receiving cTRT. Toxicities were manageable; radiation-related events were mostly grade 1–2, and no grade 5 adverse events occurred.

**Conclusion:**

The cTRT following chemoimmunotherapy was associated with a higher incidence of additional tumor shrinkage and was independently related to longer survival. Prospective randomized trials are needed to identify the optimal efficacy and patient selection criteria for cTRT practice.

## 1 Introduction

Small cell lung cancer (SCLC) is a distinct subtype of lung cancer, accounting for approximately 15% of all cases (Bray et al., 2024). It is characterized by highly aggressive behavior, including rapid growth, early metastasis, and therapeutic resistance, leading to poor survival outcomes (Wang et al., 2017). Notably, more than two-thirds of patients are diagnosed with extensive-stage SCLC (ES-SCLC) at initial presentation (Lee et al., 2023; Goyal and Sangwan, 2024), and despite standard platinum-based chemotherapy being the historical cornerstone of treatment, long-term outcomes remain extremely poor, with 5-year survival rates not exceeding 5% (Govindan et al., 2006; Canaslan et al., 2025).

The landmark IMpower133 trial presented at the 2018 World Conference on Lung Cancer (WCLC), established immunotherapy as a new standard, with atezolizumab combined with chemotherapy demonstrating significantly improved median overall survival (mOS) of 12.3 months compared to 10.3 months with chemotherapy alone (HR 0.70, 95% CI 0.54–0.91, *P* = 0.007) (Liu et al., 2021; Mansfield et al., 2020). This survival benefit was subsequently confirmed in the CASPIAN trial, in which durvalumab combined with chemotherapy further extended mOS to 12.9 months compared with 10.5 months for chemotherapy alone (HR = 0.75; 95% CI 0.62–0.91; *P* = 0.003) (Paz-Ares et al., 2019). More recently, the global phase III ASTRUM-005 trial demonstrated an even greater magnitude of benefit with serplulimab plus chemotherapy, achieving improvements in both median progression-free survival (mPFS, 5.7 vs. 4.3 months; HR = 0.48, 95% CI 0.38–0.59, *P* < 0.001) and OS (15.4 vs. 10.9 months; HR = 0.63, 95% CI 0.49–0.82, *P* < 0.001) (Cheng et al., 2025). Although these phase III studies highlight the consistent and clinically meaningful survival advances achieved with immunotherapy-based regimens in ES-SCLC, current regimens still achieve only a mPFS of 5.2–5.7 months, and most patients eventually experience local or distant relapse, underscoring the need for improved disease control. According to the Chinese Society of Clinical Oncology (CSCO) guidelines, thoracic radiotherapy (TRT) can be considered an option strategy. However, subgroup analyses from pivotal immunotherapy trials have yielded inconclusive signals regarding the added benefit of TRT, highlighting the need for further real-world evidence to clarify its optimal integration in the immunotherapy era.

The consolidative TRT (cTRT) has long been employed to enhance local tumor control, but its role after first-line therapy to improve survival outcomes for ES-SCLC in the immunotherapy era remains under debate. A retrospective clinical trial evaluated the efficacy of cTRT after first-line chemoimmunotherapy in ES-SCLC patients followed by immunotherapy maintenance. The results showed that this regimen did not significantly improve PFS (*P* = 0.93) or OS (*P* = 0.63) (Li Y. et al., 2023). Nevertheless, another retrospective analysis suggested that cTRT administered after first-line chemotherapy combined with immunotherapy was associated with significantly longer PFS compared to chemoimmunotherapy alone (1-year PFS of 61% vs. 31%, *P* < 0.001), along with a trend toward improved OS (1-year OS of 80% vs. 61%, *P* = 0.027), without a substantial increase in grade ≥3 toxicities (Longo et al., 2023).

In summary, given the limited and conflicting evidence, further studies are warranted to clarify the clinical value of cTRT in the era of chemoimmunotherapy. To address the evidence gap, we conducted a two-arm real-world comparative study to evaluate the efficacy and safety of incorporating cTRT prior to the maintenance phase in ES-SCLC patients who achieved disease control after first-line chemoimmunotherapy. Beyond conventional survival outcomes, this study specifically quantifies the incremental impact of cTRT on tumor burden reduction, aiming to clarify the depth and occurrence of further shrinkage achieved. Furthermore, acknowledging the uncertainty of real-world factors influencing cTRT selection, we analyzed the real-world decision-making logic, so as to identify potential clinical factors that may guide individualized treatment strategies and predict long-term survival benefit.

## 2 Methods

### 2.1 Study design and patients

This was a single-center, real-world, retrospective analysis of medical records from ES-SCLC patients treated at the Affiliated Hospital of Guangdong Medical University between 1 January 2018, and 31 December 2024.

The study included patients who met the following criteria: aged between 18 and 75 years of either sex; histologically or cytologically confirmed diagnosis of SCLC, with ES-SCLC defined according to the Veterans Administration Lung Study Group (VALG) system as disease extending beyond a single radiation portal that could be safely encompassed within a tolerable radiation field; Eastern Cooperative Oncology Group (ECOG) performance status of 0–2; and achieved disease control according to RECIST v1.1 after 2–6 cycles of first-line therapy of PD-1/PD-L1 inhibitor combined with platinum-etoposide chemotherapy, lastly, patients had at least one available imaging either after cTRT or during maintenance immunotherapy. All eligible patients were categorized into the cTRT group or the non-cTRT group based on whether cTRT was administered after first-line therapy. In the cTRT group, both chemotherapy and immunotherapy were suspended during the radiotherapy phase, and maintenance immunotherapy was delivered sequentially across the cohort. The cTRT was directed at the residual primary tumor after induction chemoimmunotherapy and involved-field irradiation of the mediastinal lymph node stations that were positive prior to chemotherapy. Elective nodal irradiation (ENI) was not performed. Key exclusion criteria included a history of other malignancies, autoimmune diseases, prior immune-related therapy, or the presence of interstitial lung disease (ILD) or active pulmonary fibrosis. Our institutional review board approved this study (approval number: PJKT 2024-233) and waived the requirement for informed consent due to its retrospective design. All procedures were conducted in accordance with the Declaration of Helsinki and relevant local regulations. Patient data were fully de-identified prior to analysis, and no protected information was accessible. Access to the research database was restricted to authorized study personnel only, ensuring strict protection of patient privacy.

### 2.2 Data collection and outcome assessment

Clinical characteristics collected for analysis included age, gender, smoking history, ECOG performance status, tumor stage, baseline metastatic sites, immunotherapy and chemotherapy regimens, thoracic radiotherapy details, and survival data. Serum lactate dehydrogenase (LDH) levels were collected at predefined time points, including baseline, every two treatment cycles (±7 days) during combination therapy, and additional clinically relevant time points, including within 1 week before and after cTRT, and within 1 week of disease progression.

The study focused on evaluating the efficacy and safety of cTRT. The primary endpoint was PFS, defined as the time from initiation of combined treatment therapy to the first documented disease progression or death from any cause, whichever occurred first. Secondary endpoints included OS, depth of response (DpR), and safety. OS was defined as the time from initiation of combined treatment to death from any cause. DpR was defined as the percentage reduction in the sum of target lesion diameters from baseline to the recorded tumor measurement during treatment, based on RECIST v1.1 criteria (Eisenhauer et al., 2009). The depth of further shrinkage relative to first-line chemoimmunotherapy is calculated as follows: Depth of further shrinkage (%) = (sum of diameters at subsequent assessment − sum of diameters at the end of combination therapy)/sum of diameters at the end of combination therapy × 100%. AEs were assessed and graded by senior physicians according to the Common Terminology Criteria for Adverse Events (CTCAE) version 5.0. Follow-up was conducted every 3 months during the first 2 years after cTRT, and every 4 months starting from the third year onward. Clinical status and imaging results were monitored through phone interviews and regular outpatient reviews.

### 2.3 Statistical analysis

All statistical analyses were conducted using R (Version 4.4.3), with a two-sided test and a significance level of α = 0.05. For continuous variables, normality was assessed using the Shapiro-Wilk test. Normally distributed data were presented as mean ± SD and compared using an independent-samples t-test; skewed data were described as median (range) and analyzed with the Mann-Whitney U/Kruskal-Wallis H test. Categorical variables were presented as n (%), with intergroup comparisons performed using Fisher’s exact test. Survival outcomes were estimated with the Kaplan-Meier method, and differences between groups were evaluated using the log-rank test. Inverse probability of treatment weighting (IPTW) was employed to minimize baseline confounding. Cox proportional hazards regression models identified factors associated with survival outcomes. The proportional hazards assumption was verified, and multicollinearity was excluded via the variance inflation factor (VIF <10). Logistic regression analyzed factors influencing cTRT selection, with receipt of cTRT as the outcome. AEs were coded using MedDRA 28.0 and graded according to CTCAE v5.0.

Sample size was calculated via PASS based on prior evidence (1-year PFS: 12.6% for chemoimmunotherapy (Mansfield et al., 2020); 39% for chemoimmunotherapy + sequential cTRT) (Chen et al., 2024). Considering real-world cTRT tolerance (67%) and retrospective design (Chen et al., 2024), we conservatively estimated a 40% cTRT receipt rate, setting a non-cTRT to cTRT ratio of 1.5. With α = 0.05 (two-sided), power = 0.8, and 5% annual attrition, the preset sample sizes were 54 for the non-cTRT group and 36 for the cTRT group.

## 3 Results

### 3.1 Patient baseline and treatment characteristics

From 1 January 2018, to 31 December 2024, a total of 91 patients were included in the analysis, with 39 in the cTRT group and 52 in the non-cTRT group. The characteristics of the study population were summarized in Table 1. The median age was 66 years (range, 47–83), and 82.42% were male; 57 (62.64%) patients had a history of smoking. Notably, the partial baseline disease distribution differed between groups (*P* < 0.05). The cTRT group included more patients with clinical stage of IIIB/IIIC and ECOG performance status 0–1, whereas the non-cTRT group had a heavier burden of distant metastases, including liver or adrenal involvement.

**TABLE 1:** Patients baseline characteristics.

Characteristic	Total (N = 91)	cTRT (n = 39)	Non-cTRT (n = 52)	P-value
Age (years), median (range)	66 (47,83)	64 (48,80)	66.5 (47,83)	​
Age (years), n (%)	​	​	​	0.525
<60	16 (17.58)	8 (20.51)	8 (15.38)	​
≥60	75 (82.42)	31 (79.49)	44 (84.62)	​
Gender, n (%)	​	​	​	0.233
Male	75 (82.42)	30 (76.92)	45 (86.54)	​
Female	16 (17.58)	9 (23.08)	7 (13.46)	​
Smoking history, n (%)	​	​	​	0.532
Yes	57 (62.64)	23 (58.97)	34 (65.38)	​
No	34 (37.36)	16 (41.03)	18 (34.62)	​
ECOG, n (%)	​	​	​	**0.031**
0–1	78 (85.71)	37 (94.87)	41 (78.85)	​
2	13 (14.29)	2 (5.13)	11 (21.15)	​
Clinical stage, n (%)	​	​	​	**0.001**
IIIB/IIIC	13 (14.29)	11 (28.21)	2 (3.85)	​
IVA/IVB	78 (85.71)	28 (71.79)	50 (96.15)	​
Number of metastatic lesions, n (%)	​	​	​	**0.018**
≤3	43 (47.25)	24 (61.54)	19 (36.54)	​
>3	48 (52.75)	15 (38.46)	33 (63.46)	​
Bone metastasis, n (%)	​	​	​	0.078
Yes	25 (27.47)	7 (17.95)	18 (34.62)	​
No	66 (72.53)	32 (82.05)	34 (65.38)	​
Brain metastasis, n (%)	​	​	​	0.264
Yes	19 (20.88)	6 (15.38)	13 (25)	​
No	72 (79.12)	33 (84.62)	39 (75)	​
Liver metastasis, n (%)	​	​	​	**0.007**
Yes	22 (24.18)	4 (10.26)	18 (34.62)	​
No	69 (75.82)	35 (89.74)	34 (65.38)	​
Adrenal metastasis, n (%)	​	​	​	**0.049**
Yes	12 (13.19)	2 (5.13)	10 (19.23)	​
No	79 (86.81)	37 (94.87)	42 (80.77)	​
Follow-up time, median (range)	28.43 (1.73,71.37)	28.43 (7.37,71.37)	41.03 (1.73,61.80)	​

cTRT, consolidative thoracic radiotherapy; ECOG, eastern cooperative oncology group.

Note: Bold values indicate baseline characteristics that differed significantly between the cTRT and non-cTRT groups (P < 0.05).

Regarding the first-line chemoimmunotherapy (Supplementary Table S1), there were no differences in proportions between groups, and 75.82% of patients completed 4–6 cycles. Carboplatin–etoposide was the predominant chemotherapy regimen (60.44%). Among immunotherapy agents, serplulimab was the most commonly used PD-1 inhibitor (38.46%), whereas durvalumab was the most frequently administered PD-L1 inhibitor (26.37%). The characteristics of cTRT were as shown in Supplementary Table S2. Among the patients receiving cTRT, 92.3% were treated with volume-modulated arc therapy (VMAT). The median time interval from completion of chemoimmunotherapy to initiation of cTRT was 3.0 weeks (range: 3.0–6.0 weeks). In terms of radiation dose, most patients received a total dose of 30–45 Gy (76.92%), and 31 received hyperfractionated cTRT (36–48 Gy/1.5 Gy/24–32 fractions, twice daily), 6 received conventional fractionated cTRT (50–54 Gy/1.8–2.0 Gy/25–30 fractions), and 2 received hypofractionated cTRT (50–60 Gy/2.4–2.5 Gy/20–25 fractions). Additionally, across the entire cohort, 18 patients (19.78%) underwent palliative radiotherapy to other metastatic sites during their disease course.

### 3.2 Tumor response depth and survival outcomes

**FIGURE 1:**
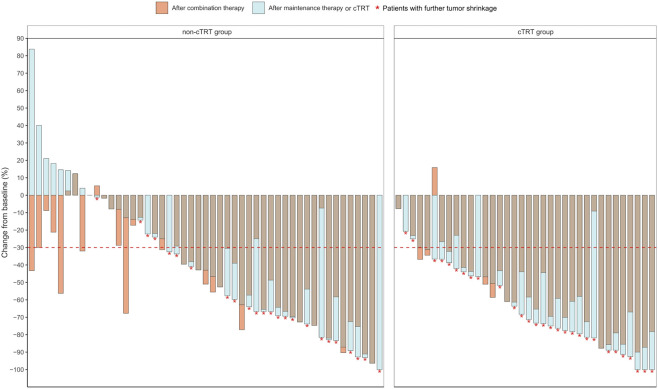
Depth of Response of Target Lesions Between cTRT and Non-cTRT Groups. This figure presents target lesion DpR for cTRT and non-cTRT groups, including DpR after combination therapy, DpR after cTRT or immune maintenance relative to baseline, and further tumor shrinkage after cTRT or immune maintenance relative to the end of combination therapy. Red bars: the DpR after combination = (diameters at the end of combination therapy − diameters at baseline)/diameters at baseline. Blue bars, Blue bars: the DpR after immune maintenance or cTRT = (diameters at subsequent assessment − diameters at baseline)/diameters at baseline. Red asterisks: Having further tumor shrinkage after immune maintenance or cTRT compared to the end of combination therapy. Depth of further shrinkage (%) = (sum of diameters at subsequent assessment − sum of diameters at the end of combination therapy)/sum of diameters at the end of combination therapy × 100%. The value < 0 was defined as further tumor shrinkage, indicated by red asterisks.

After the first-line chemoimmunotherapy, the radiologic efficacy results were in Supplementary Table S3. The objective response rate (ORR) was 79.49% (95% CI, 63.54%–90.70%) in the cTRT group and 65.38% (95% CI, 50.91%–78.03%) in the non-cTRT group, with no statistically significant difference between the two groups (*P* = 0.285). To characterize the radiologic efficacy of cTRT following chemoimmunotherapy, we evaluated the DpR at different treatment stages (Figure 1). In addition to the DpR relative to baseline, we also assessed whether patients experienced further tumor shrinkage after combination therapy, defined as the relative change in the sum of target lesion diameters at subsequent evaluations compared with the end of combination therapy. Specifically, in the cTRT group, the depth of further shrinkage was calculated as the change after cTRT relative to the end of combination therapy, while in the non-cTRT group, it was calculated as the change after maintenance immunotherapy relative to the same reference time point. Across the full cohort, more than half of patients exhibited further tumor shrinkage beyond the initial response to chemoimmunotherapy (58.24%; Supplementary Table S4). Patients receiving cTRT were substantially more likely to experience further tumor shrinkage, with 74.4% achieving additional shrinkage compared with 46.2% in the non-cTRT group (*P* = 0.007). Among patients who achieved further tumor shrinkage, the depth of further shrinkage was numerically greater in the cTRT cohort (−38.67% ± 29.06%) than in the non-cTRT cohort (−30.20% ± 28.24%), though the difference was not statistically significant (*P* = 0.208).

**FIGURE 2:**
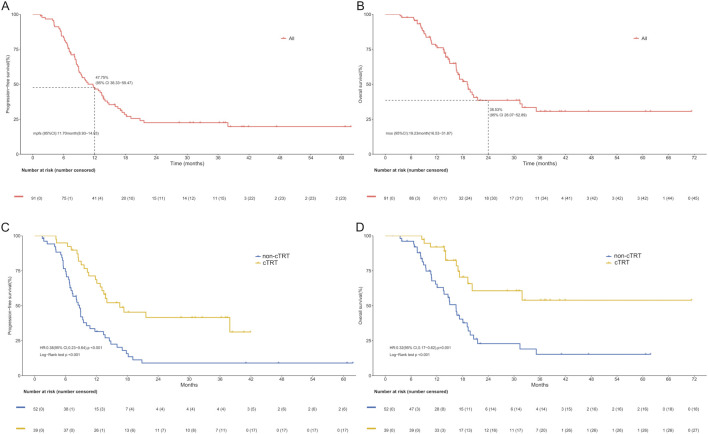
Kaplan-Meier survival curves for PFS and OS. **(A)** PFS in the overall cohort; **(B)** OS in the overall cohort; **(C)** Comparison of PFS between the cTRT and non-cTRT groups; **(D)** Comparison of OS between the cTRT and non-cTRT groups. PFS, progression-free survival; OS, overall survival; cTRT, consolidative thoracic radiotherapy.

**FIGURE 3:**
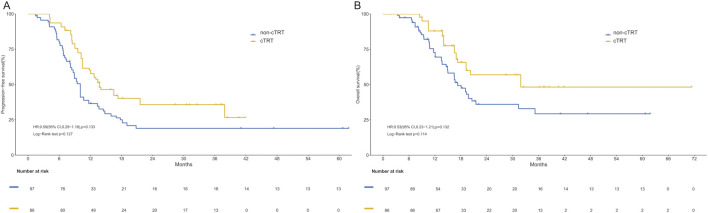
Kaplan-Meier survival curves for PFS and OS after IPTW. **(A)** Comparison of PFS between the cTRT and non-cTRT groups; **(B)** Comparison of OS between the cTRT and non-cTRT groups. PFS, progression-free survival; OS, overall survival; IPTW, inverse probability of treatment weighting; cTRT, consolidative thoracic radiotherapy.

As of the data cutoff, the median follow-up duration was 28.43 months (range, 1.73–71.37). In the overall cohort (Figures 2A,B), the mPFS was 11.70 months (95% CI, 8.93–14.03), and the mOS was 19.23 months (95% CI, 16.53–31.87), with a 2-year OS rate of 38.5%. Notably, the objective response (CR/PR vs. SD) following combination therapy did not significantly correlate with PFS or OS in our cohort (Supplementary Figures S1A,B). Patients receiving cTRT experienced a markedly prolonged PFS compared with those who did not (Figure 2C), with the mPFS being 16.53 months [95% CI, 12.73–not reached (NR)] in the cTRT group versus 8.70 months (95% CI, 7.07–10.73) in the non-cTRT group (log-rank test *P* < 0.001). A comparable trend was observed for OS (Figure 2D): the non-cTRT group had an mOS of 16.47 months (95% CI, 13.53–19.77), whereas the mOS in the cTRT group had not yet been reached (95% CI, 20.23–NR; log-rank test *P* < 0.001). Given the baseline imbalances between groups, IPTW was applied to create a weighted population with comparable characteristics (Supplementary Table S5). After IPTW adjustment, all baseline characteristics were comparable (*P* > 0.05). In the weighted cohort, the mPFS was 13.77 months (95% CI, 10.50–NR) in the cTRT group compared with 10.07 months (95% CI, 8.17–NR) in the non-cTRT group (log-rank test *P* = 0.127; Figure 3A). The IPTW-adjusted Cox model yielded a Hazard Ratio (HR) of 0.59 (95% CI: 0.29–1.18, P = 0.133). Similarly, the weighted mOS was 31.87 months (95% CI, 17.27–NR) and 17.27 months (95% CI, 13.67–NR) for the cTRT and non-cTRT groups, respectively (log-rank test *P* = 0.114; Figure 3B), with an adjusted HR of 0.53 (95% CI: 0.23–1.21, *P* = 0.132). While the survival estimates numerically favored the cTRT group, the difference did not reach statistical significance after accounting for treatment selection bias through IPTW.

### 3.3 Factors associated with survival outcomes

Since IPTW adjustment might lead to deviations from real-world clinical practice, we further conducted Cox regression analyses to identify key factors associated with survival outcomes. The results for PFS are presented in Table 2. In univariate analyses, ECOG performance status ≥2, clinical stage IVA/IVB, more than three metastatic lesions, and absence of cTRT were all associated with shorter PFS (all *P* < 0.05). After adjusting for potential confounders in the multivariate analysis, having more than three metastatic lesions remained an independent risk factor for inferior PFS (HR = 1.93, 95% CI: 1.08–3.46, *P* = 0.026), whereas receipt of cTRT was independently associated with prolonged PFS (HR = 0.54, 95% CI: 0.31–0.96, *P* = 0.036). The effects of ECOG status and clinical stage were no longer statistically significant after adjustment (all *P* > 0.05). Factors associated with OS are summarized in Table 3. Univariate findings were generally consistent with those from the PFS analyses. In multivariate analyses, receipt of cTRT emerged as the only independent factor associated with longer OS (HR = 0.45, 95% CI: 0.22–0.94, *P* = 0.033). A comprehensive comparison of HRs across unadjusted, multivariable-adjusted, and IPTW-adjusted models is provided in Supplementary Table S6.

**TABLE 2:** Univariate and multivariate Cox analyses of PFS for patients.

Characteristics	Univariate analysis	*P*	Multivariate analysis	*P*
	HR (95% CI)		HR (95% CI)	
Age (years)				
≥60 vs. <60	1.10 (0.59–2.07)	0.756	1.07 (0.56–2.08)	0.831
Gender				
Female vs. Male	0.78 (0.40–1.48)	0.441	0.96 (0.49–1.9)	0.917
Smoking history				
Yes vs. No	1.08 (0.66–1.79)	0.750	1.24 (0.73–2.1)	0.419
ECOG				
≥2 vs. 0–1	2.15 (1.16–3.98)	**0.015**	1.49 (0.77–2.89)	0.235
Clinical stage				
IVA/IVB vs. IIIB/IIIC	2.90 (1.25–6.73)	**0.013**	1.46 (0.55–3.86)	0.442
Number of metastatic lesions, n (%)				
>3 vs. ≤3	2.60 (1.56–4.32)	**<0.001**	1.93 (1.08–3.46)	**0.026**
Treatment group				
cTRT group vs. non-cTRT group	0.38 (0.23–0.64)	**<0.001**	0.54 (0.31–0.96)	**0.036**

PFS, progression-free survival; ECOG, eastern cooperative oncology group; cTRT, consolidative thoracic radiotherapy.

Note: Bold values indicate variables significantly associated with PFS in the corresponding Cox regression analyses (P < 0.05).

**TABLE 3:** Univariate and multivariate Cox analyses of OS for patients.

Characteristics	Univariate analysis	*P*	Multivariate analysis	*P*
	HR (95% CI)		HR (95% CI)	
Age (years)				
≥60 vs. <60	1.77 (0.75–4.18)	0.197	1.70 (0.70–4.14)	0.240
Gender				
Female vs. Male	0.76 (0.34–1.70)	0.502	0.91 (0.39–2.09)	0.816
Smoking history				
Yes vs. No	1.08 (0.59–1.99)	0.795	1.20 (0.62–2.33)	0.586
ECOG				
≥2 vs. 0–1	2.36 (1.12–4.95)	**0.023**	1.49 (0.66–3.33)	0.337
Clinical stage				
IVA/IVB vs. IIIB/IIIC	3.47 (1.08–11.21)	**0.037**	1.76 (0.47–6.62)	0.401
Number of metastatic lesions, n (%)				
>3 vs. ≤3	2.30 (1.25–4.22)	**0.007**	1.60 (0.81–3.16)	0.173
Treatment group				
cTRT group vs. non-cTRT group	0.32 (0.17–0.62)	**0.001**	0.45 (0.22–0.94)	**0.033**

OS, overall survival; HR, hazard ratio; ECOG, eastern cooperative oncology group; cTRT, consolidative thoracic radiotherapy.

Note: Bold values indicate variables significantly associated with OS in the corresponding Cox regression analyses (P < 0.05).

### 3.4 Factors associated with clinical selection of cTRT

In real-world clinical practice, the survival differences observed between patients who did and did not receive cTRT may be influenced not only by treatment effect but also by patient- or disease-related factors that drive treatment selection. To identify factors affecting clinicians’ decisions to administer cTRT, we performed logistic regression analyses using receipt of cTRT as the dependent variable (Table 4). In the univariate model, ECOG performance status ≥2, clinical stage IVA/IVB, more than three metastatic lesions, and liver metastasis were all associated with a reduced likelihood of receiving cTRT (*P* < 0.05). In contrast, age, sex, and smoking history showed no significant association with treatment selection. In the multivariable analysis, poor ECOG performance status (OR = 0.17, 95% CI: 0.02–0.83; *P* = 0.047) and higher clinical stage (OR = 0.16, 95% CI: 0.02–0.83; *P* = 0.046) remained independent predictors of a lower likelihood of receiving cTRT, whereas other variables did not retain statistical significance.

**TABLE 4:** Univariate and multivariate logistic analyses of the clinical selection of cTRT.

Characteristics	Univariate analysis	*P*	Multivariate analysis	*P*
	OR (95% CI)		OR (95% CI)	
Age (years)				
≥60 vs. <60	0.70 (0.23–2.11)	0.526	0.67 (0.18–2.39)	0.537
Gender				
Female vs. Male	1.93 (0.65–5.94)	0.238	1.00 (0.25–3.92)	0.995
Smoking history				
Yes vs. No	0.76 (0.32–1.80)	0.532	0.62 (0.22–1.75)	0.371
ECOG				
≥2 vs. 0–1	0.20 (0.03–0.81)	**0.046**	0.17 (0.02–0.83)	**0.047**
Clinical stage				
IVA/IVB vs. IIIB/IIIC	0.10 (0.02–0.41)	**0.004**	0.16 (0.02–0.83)	**0.046**
Number of metastatic lesions, n (%)				
>3 vs. ≤3	0.36 (0.15–0.84)	**0.019**	1.06 (0.34–3.41)	0.921
Bone metastasis				
Yes vs. No	0.41 (0.14–1.08)	0.082	0.71 (0.19–2.47)	0.590
Brain metastasis				
Yes vs. No	0.55 (0.18–1.54)	0.268	0.91 (0.25–3.13)	0.880
Liver metastasis				
Yes vs. No	0.22 (0.06–0.65)	**0.011**	0.34 (0.08–1.24)	0.119
Adrenal metastasis				
Yes vs. No	0.23 (0.03–0.93)	0.066	0.42 (0.05–2.20)	0.331

cTRT, consolidative thoracic radiotherapy; OR, odds ratio; ECOG, eastern cooperative oncology group.

Note: Bold values indicate variables significantly associated with the clinical selection of cTRT in the corresponding logistic regression analyses (P < 0.05).

### 3.5 Exploratory analyses

LDH is a widely used indicator of tumor metabolic activity and systemic tumor burden. Longitudinal comparisons revealed distinct LDH kinetics between the two groups (Supplementary Figure S2A; Supplementary Table S7). Patients in the cTRT group maintained lower and more stable LDH values with minimal fluctuation over time, whereas the non-cTRT group exhibited markedly greater variability, including intermittent elevations and wider dispersion of measurements, potentially suggesting more dynamic underlying disease activity. In within-group analyses, LDH levels in the cTRT cohort showed a significant decline following cTRT (*P* < 0.0001; Supplementary Figure S2B), and all measurements remained within the normal reference range since therapy (109–245 U/L). No comparable trend was observed in the non-cTRT group, whose LDH patterns did not demonstrate stability (Supplementary Figure S2C).

### 3.6 Safety

The toxicity profiles of both groups are summarized in Table 5. Most AEs were mild to moderate and manageable. Myelosuppression was the most frequently observed AE in both groups (28.21% in the cTRT group and 15.38% in the non-cTRT group), and only four patients in each group experienced grade 3–4. Among patients receiving cTRT, radiation esophagitis (23.08%, grade 1–2) was the second most common AE, followed by radiation pneumonitis (15.38%, grade 1–2). Notably, only one grade 3 AEs attributable to thoracic radiotherapy were observed (2.56%), and no grade 5 AEs occurred in either group.

**TABLE 5:** Safety profile of patients.

AEs	cTRT (n = 39)			Non-cTRT (n = 52)		
	Total	Grade 1–2	Grade ≥3	Total	Grade 1–2	Grade ≥3
All AEs	23 (58.97%)	20 (51.28%)	6 (15.38%)	15 (28.85%)	9 (17.31%)	6 (11.54%)
Radiation-related AEs	13 (33.33%)	13 (33.33%)	1 (2.56%)	0	0	0
Details of AEs						
Myelosuppression	11 (28.21%)	7 (17.95%)	4 (10.26%)	8 (15.38%)	4 (7.69%)	4 (7.69%)
Radiation esophagitis	9 (23.08%)	9 (23.08%)	0 (0.00%)	​	​	​
Radiation pneumonitis	6 (15.38%)	6 (15.38%)	0 (0.00%)	​	​	​
Anorexia	1 (2.56%)	1 (2.56%)	0 (0.00%)	2 (3.85%)	2 (3.85%)	0 (0.00%)
Fatigue	1 (2.56%)	1 (2.56%)	0 (0.00%)	1 (1.92%)	1 (1.92%)	0 (0.00%)
Vomiting	1 (2.56%)	0 (0.00%)	1 (2.56%)	1 (1.92%)	1 (1.92%)	0 (0.00%)
Nausea	1 (2.56%)	0 (0.00%)	1 (2.56%)	1 (1.92%)	1 (1.92%)	0 (0.00%)
Atrial Fibrillation	1 (2.56%)	1 (2.56%)	0 (0.00%)	1 (1.92%)	0 (0.00%)	1 (1.92%)
Interstitial Pneumonia	1 (2.56%)	1 (2.56%)	0 (0.00%)	1 (1.92%)	1 (1.92%)	0 (0.00%)
Upper Extremity Edema	1 (2.56%)	1 (2.56%)	0 (0.00%)	​	​	​
Hyponatremia	​	​	​	1 (1.92%)	0 (0.00%)	1 (1.92%)
Weight Loss	​	​	​	1 (1.92%)	1 (1.92%)	0 (0.00%)
Herpes Zoster	1 (2.56%)	1 (2.56%)	0 (0.00%)	​	​	​
Hand-Foot Syndrome	​	​	​	1 (1.92%)	1 (1.92%)	0 (0.00%)
Dyspnea	1 (2.56%)	1 (2.56%)	0 (0.00%)	​	​	​
Hypothyroidism	​	​	​	1 (1.92%)	1 (1.92%)	0 (0.00%)
Leukopenia	1 (2.56%)	0 (0.00%)	1 (2.56%)	​	​	​
Gastrointestinal Reaction	​	​	​	1 (1.92%)	1 (1.92%)	0 (0.00%)
Chest Tightness	1 (2.56%)	1 (2.56%)	0 (0.00%)	​	​	​
Diarrhea	​	​	​	1 (1.92%)	1 (1.92%)	0 (0.00%)
Hypersensitivity	1 (2.56%)	1 (2.56%)	0 (0.00%)	​	​	​

AEs, adverse events; cTRT, consolidative thoracic radiotherapy.

## 4 Discussion

The IMpower133 and CASPIAN trials marked a pivotal shift in the first-line management of ES-SCLC, firmly establishing the role of immunotherapy when combined with chemotherapy (Mansfield et al., 2020; Paz-Ares et al., 2019). Despite this major advancement, the addition of atezolizumab or durvalumab resulted in only a modest OS benefit of approximately 2 months and a limited PFS gain of no more than 1 month. This limited survival gain prompted the exploration of alternative or more effective PD-1/PD-L1 inhibitors in combination with chemotherapy. Several subsequent phase II/III studies evaluating agents such as serplulimab, adebrelimab, and other checkpoint inhibitors have reported different effective improvements in survival outcomes (Cheng et al., 2025; Chen et al., 2024; Kim et al., 2025). However, these incremental benefits have not fundamentally altered the natural history of ES-SCLC, as the majority of patients still experience early disease progression and rapid relapse (Kang et al., 2024). Given these challenges, there has been an enduring interest in incorporating local treatment strategies to reinforce systemic control. As early as 1999, Jeremic et al. demonstrated that cTRT, when administered following chemotherapy, significantly improved both local tumor control and survival in ES-SCLC (Jeremic et al., 1999). However, in the immunotherapy era, high-quality evidence regarding the optimal value, timing, and patient selection for cTRT remains scarce and inconclusive (Wróbel et al., 2026). Therefore, to address this unmet clinical need, we conducted this study to evaluate the efficacy and safety of cTRT administered prior to maintenance immunotherapy in ES-SCLC patients who achieved disease control following first-line chemoimmunotherapy, and to further delineate the clinical factors influencing patient selection for cTRT.

In this real-world cohort of 91 ES-SCLC patients, significant differences were observed in several baseline characteristics between the two treatment groups, including ECOG performance status, clinical stage, and number of metastatic lesions; but the chemoimmunotherapy regimen was comparable between groups. In terms of treatment response, our study demonstrated that patients receiving cTRT were markedly more likely to achieve further regression after first-line therapy (74.4% vs. 46.2%; *P* = 0.007). Although the magnitude of further shrinkage did not differ significantly between groups, the cTRT cohort showed a numerical trend toward deeper radiologic response. Regarding survival outcomes, unadjusted analyses showed that cTRT was associated with substantial and statistically significant improvements in both PFS (16.53 vs. 8.70 months; log-rank test *P* < 0.001) and OS (not reached vs. 16.47 months; log-rank test *P* < 0.001). To mitigate confounding arising from these baseline differences, IPTW adjustment was applied. It is noteworthy that while the statistical significance was attenuated in the IPTW-adjusted analysis, the direction and magnitude of the treatment effect remained highly consistent across all statistical models (Supplementary Table S6). The HRs for PFS (ranging from 0.38 to 0.59) and OS (ranging from 0.32 to 0.53) consistently favored the cTRT group, suggesting a potential clinical benefit despite the variations in *P*-values due to statistical weighting. Accordingly, we performed Cox regression to identify whether cTRT confers an independent therapeutic benefit. Both univariate and multivariate models demonstrated that cTRT was significantly associated with prolonged PFS and OS, functioning as an independent protective factor against disease progression and death. Notably, the attenuation of effect after IPTW does not deny the therapeutic value of cTRT; instead, it highlights a deeper property of real-world evidence: treatment assignment is inherently linked with prognostic determinants. In clinical practice, the decision to administer cTRT is strongly guided by these same factors—performance status, metastatic burden, and disease extent—that also impact patient survival. As a result, the unadjusted survival benefit reflects a mixture of true treatment effect and clinician-driven selection effect. Conversely, IPTW creates a hypothetical statistical scenario in which patients with poor ECOG scores or high metastatic burden are weighted as if they were equally likely to receive cTRT, leading to an overcorrection that diverges from real clinical practice. Once these clinically meaningful selection factors are stripped from the total effect, the isolated independent effect of cTRT naturally becomes smaller and statistically nonsignificant, which is expected in weighted pseudo-populations. Therefore, we conducted logistic regression to quantify clinical factors that drove its use in real-world practice. The results confirmed a distinct selection in treatment practice; clinical decision-making for cTRT was strongly shaped by patient condition. In univariate analysis, poor ECOG performance, advanced stage, and higher metastatic burden were all associated with reduced use of cTRT, while multivariable analysis confirmed ECOG performance status and clinical stage as independent determinants of treatment selection, underscoring that clinicians preferentially selected cTRT for patients with better functional status and less extensive disease. In the chemotherapy era, patient selection has been recognized as a crucial factor in determining the benefit of cTRT. Previous studies suggested that cTRT should be prioritized for patients who exhibited a favorable response to induction chemotherapy and had a limited number of metastatic lesions—particularly those with fewer than two, reflecting an oligometastatic state. And favorable initial baseline characteristics, including the absence of liver or brain metastases and preserved performance status, suggested that these patients might even tolerate higher radiation doses (45–54 Gy) with the potential for improved survival outcomes (Slotman et al., 2017; Zhang et al., 2019; Yoon et al., 2019). However, patient selection criteria in the immunotherapy era remain less clearly defined. A recent review suggested that the fundamental principles developed during the chemotherapy era, namely good performance status and lower tumor burden, may still apply to immunotherapy-based regimens (Li H. et al., 2023). This aligns with the observations from our study, in which patients with lower ECOG performance status and disease burden appeared more likely to receive and benefit from cTRT.

Biologically, these findings may reflect the profound influence of tumor burden on the immune tumor microenvironment (TME). High tumor burden is usually accompanied by a highly immunosuppressive tumor microenvironment, characterized by an accumulation of regulatory T cells, a higher proportion of M2 macrophages, and widespread cytokine dysregulation. These alterations collectively lead to a “cold” immune phenotype (Zhang et al., 2022; Savas et al., 2018). Under such conditions, radiotherapy has limited capacity to trigger adaptive or innate immune priming and, consequently, is less likely to produce meaningful synergistic effects with immune checkpoint inhibitors; in contrast, a lower tumor burden is associated with a less suppressive TME, in which local radiotherapy can more effectively stimulate systemic immune activation and potentially induce abscopal effects (Wang et al., 2019; Berg and Pietras, 2022). Although this concept is supported by preclinical and some clinical data, evidence specific to ES-SCLC remains scarce, in part because immunotherapy was introduced relatively late in this disease. To address the diminished abscopal potential in high-tumor-burden or immunosuppressive tumors, the proposed “radscopal effect” provides a new mechanistic paradigm (Barsoumian et al., 2020). Unlike traditional radiotherapy, the radscopal effect emphasizes the use of low-dose radiotherapy (LDRT, usually <5 Gy) at dose levels insufficient to induce direct DNA damage or cytotoxicity, but instead reprogram the tumor microenvironment, especially in “immune desert” tumors where the conventional abscopal effect is hard to take effect (Mavragani et al., 2016). This approach has been proposed to overcome two major limitations of traditional abscopal response strategies: the penetration of immune barriers into distant tumors and the efficient activation of systemic anti-tumor immunity. Specifically, the recent prospective LEAD trial showed encouraging efficacy of LDRT combined with durvalumab and etoposide/platinum (EP) as a first-line treatment for ES-SCLC. The trial reported a mPFS of 8.3 months (80% CI: 4.8–12.3) and a mOS of 23.6 months (80% CI: 14.1–37.6), with 1-year and 2-year OS rates of 66.7% and 50.0%, respectively (Zhang et al., 2025).

Our findings are consistent with a recent multicenter retrospective study, which reported an mPFS and mOS of 11.50 and 28.68 months in the cTRT group, respectively, with significantly reduced risks of progression (HR = 0.60, 95% CI: 0.36–0.99, *P* = 0.043) and death (HR = 0.56, 95% CI: 0.32–0.96, *P* = 0.033) (Yao et al., 2025). Furthermore, multiple retrospective studies similarly identified cTRT as an independent factor associated with improved survival (Longo et al., 2023; Cai et al., 2023; Borghetti et al., 2025; Čakš et al., 2025; Monaca et al., 2025). Several prospective cohort trials also revisited the incorporation of cTRT in ES-SCLC in the context of modern chemoimmunotherapy. Perez et al. and Welsh et al. evaluated the efficacy of cTRT, administered either sequentially or concurrently with immunotherapy following chemotherapy, in patients with ES-SCLC. The results demonstrated that this treatment strategy did not significantly improve survival outcomes, with mPFS of 4.5–6.1 months and mOS of 8.4–11.7 months (Perez et al., 2021; Welsh et al., 2020). These findings suggest that first-line chemoimmunotherapy remains the foundation for achieving prolonged survival in ES-SCLC. Another prospective phase II study investigated the efficacy and safety of a PD-L1 inhibitor, adebrelimab, in combination with chemotherapy followed by sequential TRT as first-line treatment for ES-SCLC. The results showed that in the TRT group, the mPFS was 11.3 months (95% CI, 8.3–NR), and mOS reached 22.9 months (95% CI, 20.1–NR), both significantly superior to the non-TRT group (Chen et al., 2024). Notably, the survival outcomes observed in our study appear numerically longer than those reported in this phase II trial; it is likely due to the fact that patients evaluated as having progressive disease following induction therapy were ineligible for TRT, yet their survival and adverse event data were retained in the analysis set.

LDH is widely acknowledged as a key biomarker of metabolic reprogramming and proliferative activity in malignant tumors. Elevated LDH levels are closely associated with overall tumor burden and invasive potential (Xu et al., 2021; Mezquita et al., 2018). Prior studies in first-line immunotherapy for SCLC have consistently shown that elevated LDH reflects aggressive disease biology, rapid cellular turnover, and increased glycolytic activity, and is therefore associated with poorer prognosis and inferior responses to systemic therapy, including immune checkpoint inhibitors (Zhou et al., 2025; Zhang et al., 2016; Wang et al., 2022; Mishra and Banerjee, 2019). However, limited studies have examined LDH kinetics in ES-SCLC cTRT treatment. Evidence in non-small cell lung cancer (NSCLC) suggests that longitudinal LDH kinetics may provide greater predictive value than single time-point measurements (Sung et al., 2023; Li et al., 2018). In our exploratory analysis, although LDH was not a primary endpoint, patients receiving cTRT exhibited more stable LDH trajectories throughout the treatment course, potentially indicating less fluctuation in underlying disease activity. Therefore, further prospective investigation is warranted to clarify the clinical significance of LDH dynamics in ES-SCLC and to determine whether LDH-based monitoring may have practical utility in guiding treatment selection for cTRT.

By offering real-world evidence that reflects contemporary practice, this study quantified the incremental tumor shrinkage specifically attributed to cTRT and extended beyond survival outcomes to deeply analyze and characterize the clinical factors influencing treatment selection. These results may assist in individualizing consolidative strategies and help clarify which patients are most likely to derive the greatest benefit in the immunotherapy era. However, this study had several limitations. First, we estimated a sample size of 54 patients in the non-cTRT group and 36 in the cTRT group, for a total of 90 patients, to achieve 80% power for the prespecified effect. In practice, the non-cTRT group enrolled 52 patients, while the cTRT group included 39 patients, giving an overall of 91 patients. We acknowledge that this slight shortfall may have modestly reduced statistical power, potentially limiting the ability to detect small effect sizes. This likely contributed to the non-significant P-values observed in the weighted cohort analysis. Second, as a single-center retrospective study, the generalizability of our findings is inherently limited and should be interpreted with caution. Third, as a real-world study, it inevitably involves selection biases. Including potential immortal time bias as patients in the cTRT group had to survive and maintain disease control until the initiation of radiotherapy. Finally, there was heterogeneity in the cTRT dose and fractionation schedules. While this reflects real-world clinical practice, it precludes definitive conclusions regarding the optimal cTRT patterns. To clarify the therapeutic value of cTRT more definitively with chemoimmunotherapy in ES-SCLC, prospective randomized evidence is required. The ongoing phase II/III RAPTOR trial (NCT04402788), which directly compares atezolizumab plus chemotherapy with or without cTRT, is expected to provide more robust and definitive data to guide clinical practice.

## 5 Conclusion

In this real-world study, cTRT administered after first-line chemoimmunotherapy was associated with meaningful improvements in PFS and OS among ES-SCLC patients who achieved disease control, and it remained an independent protective factor in multivariable analyses. Although the survival advantage was attenuated after adjustment for baseline imbalances, the consistent numerical trends, deeper radiologic tumor responses, and more favorable LDH kinetics collectively support a potential therapeutic role of cTRT in the contemporary immunotherapy era. Moreover, regression analyses revealed that treatment selection was strongly influenced by clinical factors, including disease stage, performance status, and metastatic burden, highlighting the importance of appropriate patient selection when considering cTRT.

## Data Availability

The original contributions presented in the study are included in the article/Supplementary Material, further inquiries can be directed to the corresponding author.
