# Supplementary material for: Efficacy and patient selection of consolidative thoracic radiotherapy following chemoimmunotherapy in ES-SCLC
Source: Front Cell Dev Biol. 2026 Jun 11;14:1850317. doi: 10.3389/fcell.2026.1850317 (PMC13293915; doi:10.3389/fcell.2026.1850317)
Supplement: Supplementary file 1 [file DataSheet1.docx]

Supplementary Material

# Supplementary Figure

**Supplementary Figure 1. Kaplan-Meier survival curves for PFS and OS.**

**
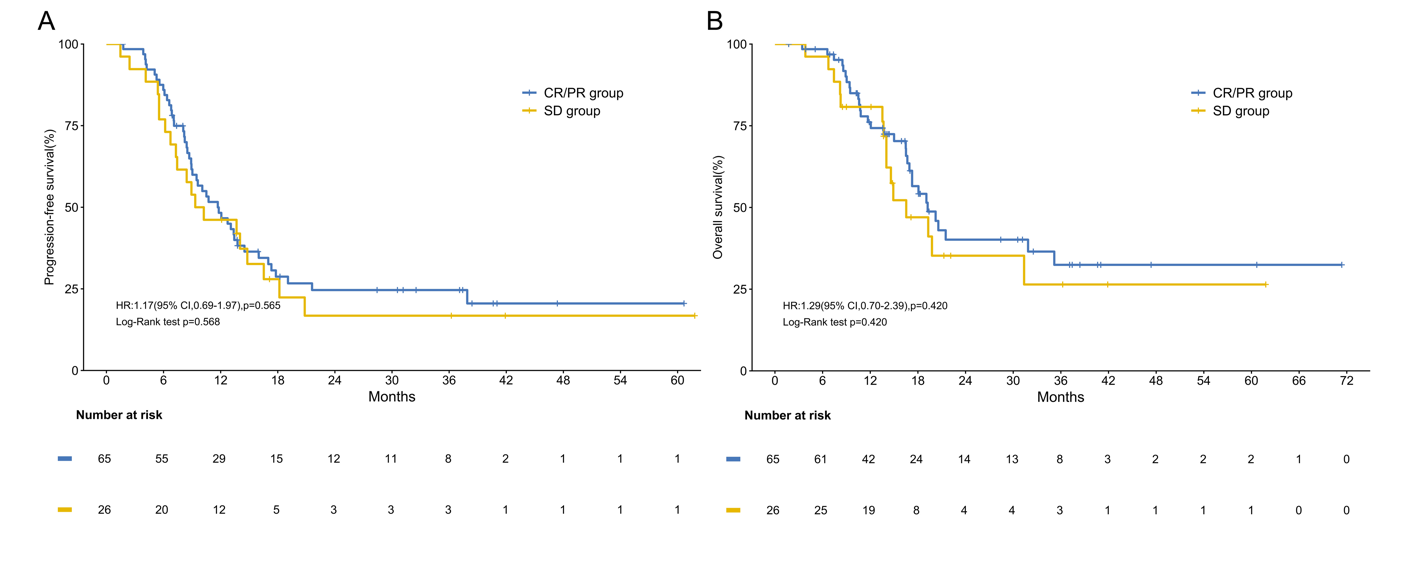
**

(A) Comparison of PFS between the CR/PR and SD; (B) Comparison of OS between the CR/PR and SD. PFS, progression-free survival; OS, overall survival; ORR, objective response rate

**Supplementary Figure 2. Analysis of LDH levels within and between groups in longitudinal comparison.**


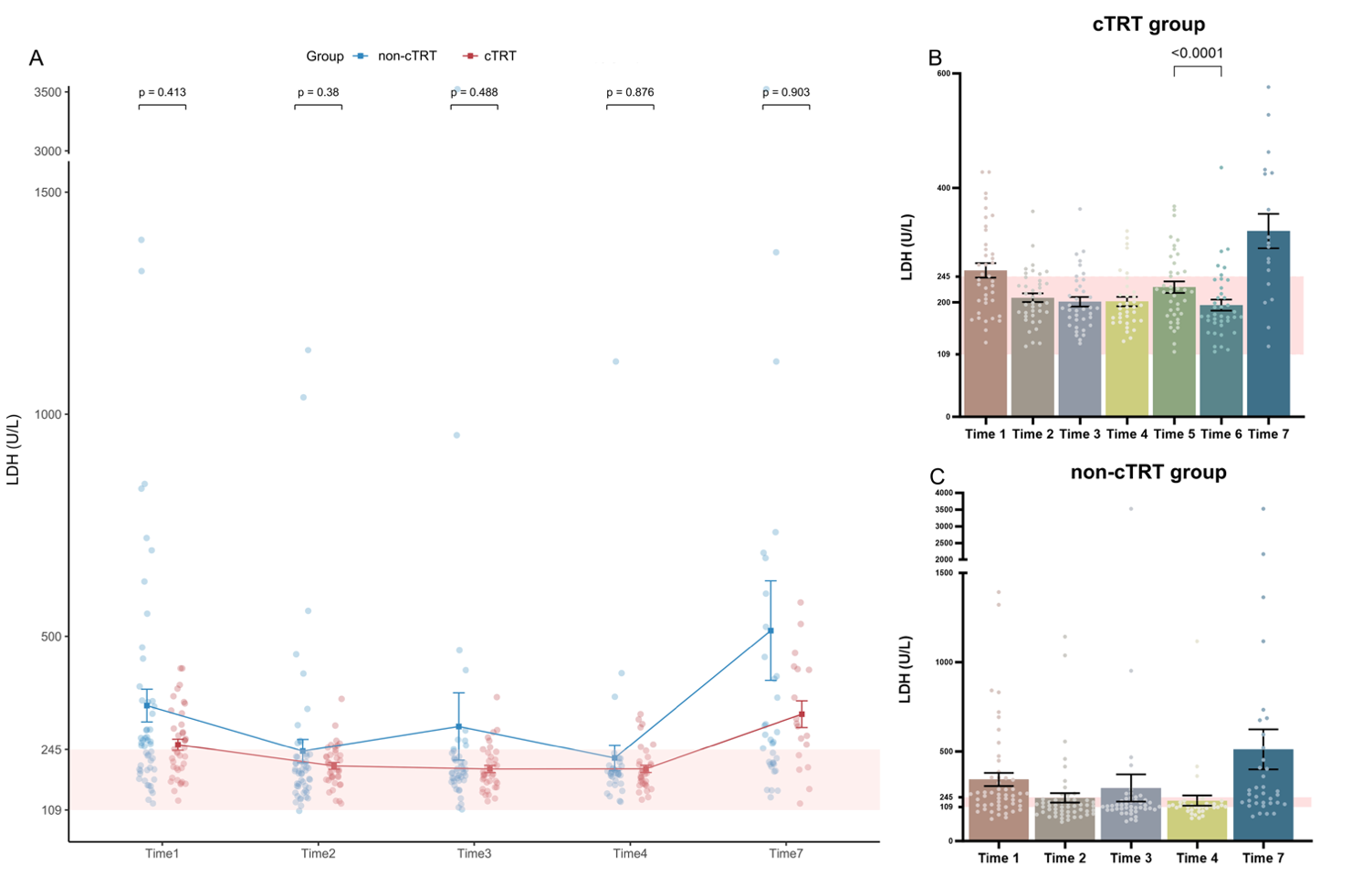


(A) Comparison of changes in LDH between the two groups; (B) Comparison of changes in LDH in the cTRT group; (C) Comparison of changes in LDH in the non-cTRT group.

Time 1: LDH at baseline; Time 2: LDH after 2 cycles of combination therapy; Time 3: LDH after 4 cycles of combination therapy; Time 4: LDH after 6 cycles of combination therapy; Time 5: LDH before cTRT; Time 6: LDH after cTRT; Time 7: LDH at disease progression. The red box shadow indicates the normal reference range of LDH (109–245 U/L). LDH, lactate dehydrogenase; cTRT, consolidative thoracic radiotherapy

# Supplementary Tables

**Supplementary Table 1. Detailed Characteristics of Chemotherapy and Immunotherapy**

| Characteristic | Total (N=91) | cTRT (n=39) | Non-cTRT (n=52) | P-value |
| --- | --- | --- | --- | --- |
| Number of chemo-immunotherapy cycles, n (%) |  |  |  | 0.480 |
| <4 | 22 (24.18) | 8 (20.51) | 14 (26.92) |  |
| 4-6 | 69 (75.82) | 31 (79.49) | 38 (73.08) |  |
| Chemotherapy regimen during combination therapy, n (%) |  |  |  | 0.658 |
| Etoposide + carboplatin | 55 (60.44) | 25 (64.1) | 30 (57.69) |  |
| Etoposide + lobaplatin | 18 (19.78) | 6 (15.38) | 12 (23.08) |  |
| Etoposide + cisplatin | 18 (19.78) | 8 (20.51) | 10 (19.23) |  |
| Immunotherapy agent during combination therapy, n (%) |  |  |  | 0.628 |
| PD-1 | 47 (51.65) | 19 (48.72) | 28 (53.85) |  |
| Camrelizumab | 1 (1.1) |  | 1 (1.92) |  |
| Pucotenlimab | 1 (1.1) | 1 (2.56) |  |  |
| Serplulimab | 35 (38.46) | 13 (33.33) | 22 (42.31) |  |
| Toripalimab | 2 (2.2) | 1 (2.56) | 1 (1.92) |  |
| Tislelizumab | 8 (8.79) | 4 (10.26) | 4 (7.69) |  |
| PD-L1 | 44 (48.35) | 20 (51.28) | 24 (46.15) |  |
| Adebrelimab | 6 (6.59) | 5 (12.82) | 1 (1.92) |  |
| Atezolizumab | 11 (12.09) | 6 (15.38) | 5 (9.62) |  |
| Benmelstobart | 2 (2.2) | 1 (2.56) | 1 (1.92) |  |
| Durvalumab | 24 (26.37) | 7 (17.95) | 17 (32.69) |  |
| Envafolimab | 1 (1.1) | 1 (2.56) |  |  |
| Total number of immunotherapy cycles, n (%) |  |  |  |  |
| <6 | 21 (23.08) | 4 (10.26) | 17 (32.69) |  |
| 6-34 | 65 (71.43) | 33 (84.62) | 32 (61.54) |  |
| ≥34 | 5 (5.49) | 2 (5.13) | 3 (5.77) |  |

cTRT, consolidative thoracic radiotherapy; PD-1, programmed cell death protein 1; PD-L1, programmed death-ligand 1

**Supplementary Table 2. Detailed Characteristics of Radiotherapy**

| Characteristic | Total (N=91) | cTRT (n=39) | Non-cTRT (n=52) |
| --- | --- | --- | --- |
| Modality of consolidative thoracic radiotherapy, n (%) |  |  |  |
| VMAT | 36 (39.56) | 36 (92.31) |  |
| 3D-CRT | 2 (2.2) | 2 (5.13) |  |
| IMRT | 1 (1.1) | 1 (2.56) |  |
| Total dose of cTRT, n (%) |  |  |  |
| 30-45Gy | 30 (32.97) | 30 (76.92) |  |
| >45Gy | 9 (9.89) | 9 (23.08) |  |
| Fractionation pattern, n (%) |  |  |  |
| Hyperfractionation | 31 (34.07) | 31 (34.07) |  |
| Conventional fractionation | 6 (6.60) | 6 (6.60) |  |
| Hypofractionation | 2 (2.20) | 2 (2.20) |  |
| PCI, n (%) |  |  |  |
| Yes | 13 (14.29) | 9 (23.08) | 4 (7.69) |
| No | 78 (85.71) | 30 (76.92) | 48 (92.31) |
| Palliative radiotherapy to other metastatic sites, n (%) |  |  |  |
| Yes | 18 (19.78) | 6 (15.38) | 12 (23.08) |
| No | 73 (80.22) | 33 (84.62) | 40 (76.92) |

cTRT, consolidative thoracic radiotherapy; VMAT, volume-modulated arc therapy; 3D-CRT, three-dimensional conformal radiotherapy; IMRT, intensity-modulated radiotherapy; PCI, prophylactic cranial irradiation

**Supplementary Table 3. Best overall response to chemoimmunotherapy in cTRT and Non-cTRT Groups.**

| Characteristic | Total (N = 91) | cTRT (n = 39) | Non-cTRT (n=52) | P-value |
| --- | --- | --- | --- | --- |
| Best overall response |  |  |  | 0.285 |
| CR | 6 (6.59%) | 3 (7.69%) | 3 (5.77%) |  |
| PR | 59 (64.84%) | 28 (71.79%) | 31 (59.62%) |  |
| SD | 26 (28.57%) | 8 (20.51%) | 18 (34.62%) |  |
| ORR (CR+PR) | 65 (71.43%) | 31 (79.49%) | 34 (65.38%) |  |
| 95% CI | 61.00%-80.41% | 63.54%-90.70% | 50.91%-78.03% |  |

CR, complete response; PR, partial response; SD, stable disease

**Supplementary Table 4. Depth of Response of Target Lesions Between cTRT and Non-cTRT Groups.**

| Characteristic | Total (N=91) | cTRT (n=39) | Non-cTRT (n=52) | P-value |
| --- | --- | --- | --- | --- |
| Patients with further shrinkage, n (%) | 53 (58.24) | 29 (74.36) | 24 (46.15) | **0.007** |
| Depth of further shrinkage (%), mean ± SD | -34.83±28.73 | -38.67±29.06 | -30.2±28.24 | 0.208 |

Depth of further shrinkage (%) = (sum of diameters at subsequent assessment − sum of diameters at the end of combination therapy) / sum of diameters at the end of combination therapy × 100%; cTRT, consolidative thoracic radiotherapy

**Supplementary Table 5. Baseline characteristics of patients before and after IPTW**

| Characteristic | Without IPTW | | | With IPTW | | |
| --- | --- | --- | --- | --- | --- | --- |
|  | cTRT (n = 39) | Non-cTRT (n=52) | P-value | cTRT (n = 85.97) | Non-cTRT (n = 96.98) | P-value |
| Age (years), n (%) |  |  | 0.525 |  |  | 0.709 |
| <60 | 8 (20.51) | 8 (15.38) |  | 14.62 (17.00) | 13.67 (14.09) |  |
| ≥60 | 31 (79.49) | 44 (84.62) |  | 71.35 (83.00) | 83.32 (85.91) |  |
| Gender, n(%) |  |  | 0.233 |  |  | 0.726 |
| Male | 30 (76.92) | 45 (86.54) |  | 70.56 (82.08) | 75.71 (78.06) |  |
| Female | 9 (23.08) | 7 (13.46) |  | 15.40 (17.92) | 21.28 (21.94) |  |
| Smoking history, n (%) |  |  | 0.532 |  |  | 0.556 |
| Yes | 23 (58.97) | 34 (65.38) |  | 32.78 (38.13) | 44.41 (45.79) |  |
| No | 16 (41.03) | 18 (34.62) |  | 53.19 (61.87) | 52.58 (54.21) |  |
| ECOG, n (%) |  |  | **0.031** |  |  | 0.644 |
| 0-1 | 37 (94.87) | 41 (78.85) |  | 78.28 (91.05) | 84.46 (87.09) |  |
| ≥2 | 2 (5.13) | 11 (21.15) |  | 7.69 (8.95) | 12.52 (12.91) |  |
| Clinical stage, n (%) |  |  | **0.001** |  |  | 0.707 |
| IIIB/IIIC | 11 (28.21) | 2 (3.85) |  | 13.52 (15.73) | 19.67 (20.28) |  |
| IVA/IVB | 28 (71.79) | 50 (96.15) |  | 72.45 (84.27) | 77.31 (79.72) |  |
| Number of Metastatic Lesions, n (%) |  |  | **0.018** |  |  | 0.923 |
| ≤3 | 24 (61.54) | 19 (36.54) |  | 45.47 (52.89) | 50.10 (51.66) |  |
| >3 | 15 (38.46) | 33 (63.46) |  | 40.50 (47.11) | 46.88 (48.34) |  |

IPTW, inverse probability of treatment weighting; ECOG, Eastern Cooperative Oncology Group; cTRT, consolidative thoracic radiotherapy

**Supplementary Table 6. Comparison of HRs for cTRT versus non-cTRT group across unadjusted, multivariable, and IPTW-adjusted models**

|  | PFS | | OS | |
| --- | --- | --- | --- | --- |
| Model | HR (95% CI) | P | HR (95% CI) | P |
| Unadjusted Cox | 0.38 (0.23-0.64) | <0.001 | 0.32 (0.17-0.62) | 0.001 |
| Multivariable Cox | 0.54 (0.31-0.96) | 0.036 | 0.45 (0.22-0.94) | 0.033 |
| IPTW-adjusted Cox | 0.59 (0.29-1.18) | 0.133 | 0.53 (0.23-1.21) | 0.132 |

HR, hazard ratios; cTRT, consolidative thoracic radiotherapy; PFS, progression-free survival; OS, overall survival

**Supplementary Table 7. Analysis of LDH levels between groups in longitudinal comparison.**

|  | | | cTRT (n=39) | | Non-cTRT (n=52) | | | |  |
| --- | --- | --- | --- | --- | --- | --- | --- | --- | --- |
|  | N | Mean ± SE | Mean ± SD | Median (rang) | N | Mean ± SE | Mean ± SD | Median (rang) | P-value |
| Time 1 | 39 | 255.81±12.53 | 255.81±78.26 | 239.50 (130.00,427.70) | 52 | 343.98±36.88 | 343.98±265.94 | 258.65 (123.60,1392.80) | 0.413 |
| Time 2 | 39 | 208.15±7.64 | 208.15±47.69 | 199.00 (123.40,359.00) | 52 | 241.44±26.44 | 241.44±190.68 | 196.10 (107.00,1144.30) | 0.380 |
| Time 3 | 37 | 201.08±8.28 | 201.08±50.38 | 190.00 (128.50,363.00) | 45 | 296.90±75.81 | 296.90±508.57 | 195.00 (110.30,3523.90) | 0.488 |
| Time 4 | 35 | 201.45±8.27 | 201.45±48.92 | 191.50 (132.10,324.70) | 34 | 225.89±28.67 | 225.89±167.16 | 192.20 (127.30,1118.50) | 0.876 |
| Time 5 | 39 | 226.60±10.07 | 226.60±62.90 | 218.50 (113.50,368.00) |  |  |  |  |  |
| Time 6 | 39 | 195.22±9.60 | 195.22±59.92 | 176.00(113.70,435.50) |  |  |  |  |  |
| Time 7 | 18 | 324.65±30.00 | 324.65±127.30 | 300.35(123.40,576.20) | 35 | 512.77±112.06 | 512.77±662.95 | 267.30(137.50,3523.90) | 0.903 |

Time 1: LDH at baseline; Time 2: LDH after 2 cycles of combination therapy; Time 3: LDH after 4 cycles of combination therapy; Time 4: LDH after 6 cycles of combination therapy; Time 5: LDH before cTRT; Time 6: LDH after cTRT; Time 7: LDH at disease progression.
LDH, lactate dehydrogenase; cTRT, consolidative thoracic radiotherapy
